# Improving care for cancer-related and other forms of lymphoedema in low- and middle-income countries: a qualitative study

**DOI:** 10.1186/s12913-022-07840-7

**Published:** 2022-04-08

**Authors:** Eric Torgbenu, Tim Luckett, Mark Buhagiar, Cecilia Mauricio Requena, Jane L. Phillips

**Affiliations:** 1 IMPACCT (Improving Palliative, Aged and Chronic Care Through Clinical Research and Translation), Faculty of Health, University of Technology Sydney NSW, Sydney, Australia; 2 School of Allied Sciences, Department of Physiotherapy and Rehabilitation Sciences, University of Health and Allied Sciences, Ho, Ghana; 3 Catholic Diocese of Parramatta, Parramatta, NSW Australia; 4 HammondCare, Braeside Hospital, Prairiewood, NSW Australia; 5 School of Nursing, Queensland University of Technology, Brisbane, Australia

**Keywords:** Lymphoedema, Current practice, Low- and middle-income countries, Qualitative research

## Abstract

**Background:**

Lymphoedema is a common, distressing and debilitating condition that can be related to cancer and its treatment or other conditions. Little is known about current practices in the diagnosis, assessment and management of lymphoedema in low- and middle-income countries (LMIC).

**Aim:**

To describe current practices in diagnosing, assessing and managing cancer-related and other forms of lymphoedema in LMIC, and related barriers and facilitators.

**Methods:**

An exploratory-descriptive qualitative study. Participants were lymphoedema experts or health care professionals identified via published lymphoedema papers and professional organizations respectively. Sampling was purposive to ensure a diversity of perspectives and experience. Data collection was via semi-structured telephone/video interviews, and questions canvassed participants’ experiences and perceptions of lymphoedema care in LMIC. Interviews were audio-recorded and transcribed verbatim. Analysis proceeded via inductive coding before mapping codes to the World Health Organization’s (WHO) Innovative Care for Chronic Conditions Framework.

**Results:**

Nineteen participants were interviewed, most of whom were physiotherapists (*n* = 11). Ten participants worked permanently in a LMIC, while the remainder were based in a high-income country (HIC) and had been involved in initiatives to improve lymphoedema care across multiple LMIC. Participants indicated that management of cancer versus non-cancer related lymphoedema was similar, but that pathways to care were more straight-forward for those receiving cancer care, leading to earlier diagnosis. Key facilitators to optimizing lymphoedema care in LMIC included: 1) joining forces to overcome lymphoedema-related stigma; 2) building workforce capabilities; and 3) partnering with patients and families to support self-management. Ideas for building workforce included developing health professional knowledge, supporting a commitment to multidisciplinary team care, and adapting HIC guidelines for lymphoedema care to LMIC. Partnering with patients and families to support self-management involved following the person-centred approach, establishing clear communication, promoting adherence to management, adapting management to available resources, and involving patient family and friends in lymphoedema care.

**Conclusion:**

Raising community and health professional awareness regarding lymphoedema and its management is a key first step to improving care outcomes. Resources for clinicians and patients/families developed for lymphoedema care in HIC need to be adapted for low resource settings.

**Supplementary Information:**

The online version contains supplementary material available at 10.1186/s12913-022-07840-7.

## Background

Lymphoedema is often referred to as the “forgotten complication” because it can be mistaken for oedema, venous insufficiency or heart disease [1–3]. This chronic and debilitating condition results from impaired lymphatic drainage in the presence of normal capillary function [4]. While primary lymphoedema is often linked with an inherited autosomal dominant gene, secondary lymphoedema is acquired through damage or obstruction of the lymphatic system caused by recurrent infections, or a consequence of malignancy, cancer treatment, trauma, or obesity [4]. Regardless of the cause, lymphoedema is characterised by swelling, heaviness, regular skin infections, discomfort, pain, functional impairment and reduced quality of life [5, 6].

Globally, over 200 million people are affected by lymphoedema, with the majority of the burden borne by people in LMIC [7]. Some 67 million (34%) people living in LMIC suffer from lymphatic filariasis, a tropical parasitic disease making it the most prevalent cause of lymphoedema in LMIC [8]. Efforts to combat lymphatic filariasis differ from those for lymphoedema from other causes in that they primarily involve mass antifilarial drug administration [8, 9]. Estimates of cancer-related lymphoedema incidence and prevalence vary widely even among HIC, where the vast majority of research has been conducted [10, 11]. A point prevalence estimation of lymphoedema in the LIMPRINT study of HIC reported that the condition is very prevalent with over 38% (ranging from 11.1 to 57.1%) of patients admitted developing chronic oedema despite the cause of initial hospitalization [12]. A recent systematic review of LMIC identified an even wider variability in the prevalence of cancer-related lymphoedema, ranging from 0.4% to 92.5% (arm) and 7.0% to 13.0% (leg) [13]. Such variation suggests that a lack of a standardised approach for diagnosing and measuring lymphoedema may be among the barriers to care [13].

Once lymphoedema develops, there is no known cure and management is focussed on reducing oedema and other symptoms and restoring function [14, 15]. A wrong or delayed diagnosis affects the management of the condition in ways that greatly impact on the person’s physical and psychosocial health.

Little is known about how lymphoedema care is currently provided in LMIC, including the degree to which care for cancer-related lymphoedema varies from that offered to lymphoedema from other common causes, including lymphatic filariasis. A better understanding of current practice and related barriers and facilitators is required as the first step for improving care and outcomes.

### Aim

To describe current practices in diagnosing, assessing and managing cancer-related and other forms of lymphoedema in LMIC, and related barriers and facilitators.

## Design and methods

### Study design

A qualitative study involving a series of semi-structured interviews (‘interviews’) was conducted between November 2020 and May 2021, While data collection occurred during the COVID-19 pandemic, participants were asked to comment on lymphoedema care prior to COVID-19 to increase generalisability to care post-pandemic. This was to ensure the study findings were not limited to treatment during the pandemic only but reflected lymphoedema care more generally before and after the pandemic.

The consolidated criteria for reporting qualitative research (COREQ) was used to guide the reporting of this study [16].

### Participants and setting

Participants were included if they met one of the following criteria: a) an expert on the diagnosis, assessment and/or management of cancer-related and other forms of lymphoedema as denoted by authorship on a relevant journal article and/or b) a health professional or other person who has been involved in providing care for cancer-related and other forms of lymphoedema in a LMIC in a paid or volunteer capacity. Participants also had to be able to give written informed consent and complete the interview in either English or Spanish. We chose Spanish because it ranks highest among languages other than English when considering number of speakers in combination with number of countries worldwide.

### Recruitment

Participants were sampled using two non-probabilistic methods: purposive and snowballing methods. A purposive sampling using a snowball recruitment strategy was used to maximize the recruitment [17]. Initial approach was made via email invitation to authors of relevant journal articles identified by a systematic review of lymphoedema studies in LMIC [13], personal and professional networks, and through organizations with an interest in lymphoedema care in LMIC. Targeted journal authors were all authors of studies carried out in LMIC (see Supplementary Table 1 for list). Participants were encouraged to pass on information about the study and the team’s contact details to other people in their networks who might be eligible. This recruitment strategy was designed to capture a wide range of experts in the area, including participants with varied disciplines and experience across different LMIC.

‘Information power’—defined as the amount and relevance of information gathered from each participant [18]—was used to determine sample size.

### Data collection

The interviews were conducted by ET (a male physiotherapist with over 8 years of clinical experience in Ghana who had undertaken training in qualitative research) with assistance from a team with relevant expertise in qualitative research: TL a male speech pathologist, academic and social scientist; JLP, a female cancer/palliative care clinical academic nurse; and MB, a male physiotherapist and expert in cancer-related lymphoedema. Interviews in Spanish involved CMR as interpreter, a female physiotherapist from Peru working in palliative care in Australia. Interviewers did not know the interviewees prior to the interviews.

An interview guide informed by the literature [13] was developed (Refer to Table 1).

**Table 1:** Semi-structured interview guide

Questions for experts identified through the literature
● Please tell me about projects that you have been involved in regarding lymphoedema care in a low- and middle-income countries (LMIC)
*Prompts for each project:*
● Where was the project?
● What did it involve?
● What was your role?
● What were the main findings/outcomes of this project?
● Can you tell me what worked well for the project?
**Questions for clinicians involved in lymphoedema care**
● Can you tell me about usual care for lymphoedema in your experience?
*Prompts:*
● How is lymphoedema diagnosed?
● How is lymphoedema assessed?
● How is lymphoedema managed?
● How do people access services to help their lymphoedema?
● Which health professionals and others are involved?
● Are people with lymphoedema or their carers given information or involved in managing their condition?
● Do you use any guidelines?
● Do you have access to experts or networks who you can go for to seek advice?
**General questions for both groups**
● What difference (if any) is there between care for cancer-related lymphoedema versus lymphoedema from other causes?
● For your perspective, what are the biggest issues concerning the management of lymphoedema?
*Prompts:*
● What are the barriers for lymphoedema care?
● What are the facilitators for lymphoedema care?
● Is there anything else that you think might be helpful for us to think about on this topic?

Interviews were audio-recorded, with transcripts imported into NVivo V.12 (QSR International) for management. Field notes were taken to supplement the audio recording.

### Research team

The research team comprised of three experienced researchers (TL, JLP, and MB), a clinical physiotherapist (CMR), and a doctoral degree student (ET). All interviews were conducted by ET. Participants were informed at the start of the interviews about ET’s background as a trained physiotherapist who has practiced in a LMIC. There were no established relationship between the researcher and the participants before the commencement of the interviews.

### Data analysis

Initial line-by-line coding was used to allocate descriptive labels inductively [19]. One researcher (ET) abstracted these codes throughout the data analysis into broader categories, with review and discussion by other team members (TL, JP, and MB). While an inductive approach was deemed useful to ensure that codes were grounded in the data and to capture new insights, it was also considered necessary to build on existing theory and evidence. In the next stage of analysis, then, ET carried out deductive coding of the descriptive categories against the WHO Innovative Care for Chronic Conditions (ICCC) Framework, which divides factors influencing care for chronic conditions into “community” and “health care organization” building blocks, within an over-arching “policy environment” [20]. The “policy environment” is responsible for: promoting consistent financing; strengthening partnerships; supporting legislative frameworks; integrating policies; providing leadership and advocacy; and developing and allocating human resources. The “health care organization” component is comprised of: promoting continuity and coordination; encouraging quality through leadership and incentives; organizing and equipping health care teams; using information systems; and supporting self-management and prevention. The “community” component consists of: raising awareness and reducing stigma; encouraging better outcomes through leadership and support; mobilising and coordinating resources, and delivering complementary services to ensure better outcomes for chronic care conditions. Coding against the ICCC Framework was distilled via review and discussion with the other team members to diversify and enrichen interpretation. In reporting the findings, quotes are used to illustrate themes and enable confirmability.

Interviewees were sent a summary of emerging themes and given the opportunity to verify or disagree before they were finalised.

## Results

### Participant characteristics

Of the 38 people approached directly, 19 (50% response rate) participated in the study. Reasons for non-participation included a ‘bounce’ or non-response to emails or lack of time due to workload (heightened during the pandemic). Most participants were female (*n* = 14) and worked as physiotherapists (*n* = 11). Other participants included physicians (*n* = 2), an occupational therapist, a dermatologist, an epidemiologist, an oncology nurse, a nurse consultant, and a wound nurse consultant. Ten participants were working permanently in a LMIC while the remainder were based in a HIC (Australia, Canada, Japan, USA, and UK) but had been involved in initiatives to improve lymphoedema care across multiple LMIC. The nine participants who were not living in LMICs had a minimum of five years’ experience of working in a LMIC, many in more than one country. Many of the initiatives they worked on were largely driven by local people. Most of them used their influence in sourcing funding and building the local healthcare teams’ capabilities.

Participants’ experience of lymphoedema from various causes differed according to the country they worked in and their role. Participants (*n* = 6) working in community health centres in Haiti, Fiji, Cook Islands, Bangladesh, and China were more familiar with non-cancer related lymphoedema caused by filariasis, podoconiosis, idiopathy or trauma, whereas participants (*n* = 8) with experience in Egypt, Zambia, Ghana, Lebanon, India, and Peru worked in the cancer setting and mostly dealt with breast-cancer related lymphoedema secondary to treatment. The remainder (*n* = 5) had a mixed experience of working in both cancer and non-cancer settings, and cared for people living with lymphoedema caused by cancer and other causes.

The median length of interviews was 42 min (range 27 to 66 min).

### Themes

Emerging from the data were three themes, as described below.

### Cancer versus non-cancer related lymphoedema: different pathways, same care

Participants with experience across causes of lymphoedema indicated that people with cancer-related lymphoedema tended to be identified earlier because of regular check-ups when accessing radiotherapy and chemotherapy. Unfortunately, people with lymphoedema due to other causes tended to present later, reducing the effectiveness of management.

*“A lot of these patients [with non-cancer related lymphoedema], when they do present, it's a lot of times in a very late-stage, very emergent situation. And it's really difficult to treat at that time, and resource-intensive, as you're aware” (Participant 2, HIC Physiotherapist, reporting on lymphoedema in Haiti and India).*

Participants suggested that people with cancer-related lymphoedema were more likely to recognize signs and symptoms, including swelling and heaviness, and sought medical advice that facilitated lymphoedema care.

*“I've spoken with patients who realized the swelling, contact their doctors and ask them for solutions. And some of them were able to refer lymphoedema services” (Participant 1, Physiotherapy, Ghana).*

By comparison, people with lymphoedema from non-cancer causes were said to have minimal knowledge regarding their condition. This was especially true of filarial lymphoedema, which showed no symptoms at early stages.

*“When you work in lymphatic filariasis is very challenging because the people who are infected have no idea, there's really very little symptom to actually being infected with the parasites. But what they do see is people who come to their office with lymphoedema and hydrocele seeking care” ”(Participant 13, HIC Epidemiologist, reporting on lymphoedema in sub-Saharan African, Pacific Islands, Brazil, Dominican Republic, Guyana and Haiti and South-East Asia).*

Unlike lymphoedema from other causes, filarial lymphoedema was said to require a laboratory test to confirm and treatment with medication.

*“…but 90% of the patients are diagnosed with filariasis and they are taking regular pills for filariasis. Not even of course the test is negative. So patients do these tests and I have patients telling me that they have this medication prescribed by their doctors” (Participant 6, Physiotherapist, Egypt).*

However, once the infection was treated, participants indicated that patients with filarial lymphoedema were referred for ongoing assessment and management similar to lymphoedema from other causes. Regardless of the underlying cause, participants indicated that similar barriers and facilitators impacted on the lymphoedema care.

### Barriers and facilitators to lymphoedema care

Barriers and facilitators were identified in relation to most ICCC Framework components (Refer Table 2). Participants especially emphasised three key facilitators, namely: 1) joining forces to overcome lymphoedema related stigma; 2) building workforce lymphoedema capabilities; and 3) partnering with patients and families to support self-management.

**Table 2:** Themes and sub-themes following coding of initial inductive codes against the World Health Organization’s Innovative Care for Chronic Conditions Framework

ICCCF Domains	Themes	Sub-themes	Exemplar comments from participants
**1. Positive policy environment level**			
Develop and allocate human resources	Too little emphasis placed on integrating lymphoedema care into health workforce education and training	Lymphedema care not embedded into undergraduate physiotherapy curricula	*“In my training lymphoedema care was not really covered. It was not. And I did not really come across it during my time of training in the first few years when I started work. It was when I was posted to this unit that I started seeing a lot of them and then I had to learn to be able to manage them. So I learned from my colleague. It was a learning process for both of us, so we had to find other sources to learn from” (Participant 1, Physiotherapist, Ghana)*
			*“So we have this one year, every year training programs, certificate training programs. So they come in and train the physios and train the occupational therapists. We train them for 10 days in diagnosis and management, all sort of lymphedema bandaging, how to take a measurement for sleeves, how to order sleeves and all these things” (Participant 10, Physiotherapist, India)*
		The demand for lymphoedema care outstrips supply	*“So it's the human resource, and because there are only two of us together with other cases we see, sometimes it's a bit of a challenge having to spend a lot of time and then… So we are now having to see a lot of lymphedema cases in a day just to be able to make time for other cases” (Participant 1, Physiotherapist, Ghana)*
			*“I think sometimes the treatment can be discontinued because they go back to their villages. And there's only one nurse in the village for many, many people. So some places there was one nurse for 500 people. So then the time of the nurse, and they're not all, they don't all work the same hours that we would work, for example” (Participant 17, Wound nurse consultant, Australia)*
Integrate policies	Bureaucratic health policies	Cumbersome and inefficient healthcare systems	*“So most of these countries, they have what, like we would say the Ministry of Health or the Department of Health and everything goes through that. So you are a hospital. You want to buy some bandages, you have to put a requisition in. The bandage goes to this… The requisition goes to this man, this man looks at it. He sends it to this man who looks at it, who then sends it to the Ministry of Health to sign off on it and pay for it. And then it will come back again. So a very tedious process” (Participant 17, Wound nurse consultant, Australia)*
	Under recognition of lymphoedema as a clinical care priority		*“I mean because obviously, if you were providing care, and you don't recognize that this is a problem with the lymphatic system, and that you don't look at the strategies that you need to deal with it, you're not going to advocate with funders to provide the coverage for the cost of what you may need to do to try and help people with this problem” (Participant 19, Medical Physician, Canada)*
Financial Support	Too many other funding priorities leaves lymphoedema care in need of financial support		*“Some of us (countries) have more resources to do it and some of us have a lot less but, it's still struggling with how you (Government) support these chronic problems in a model that is sustainable financially. That's part of the problem and the gap and the difficulty is finding funding for that, because there's just so many funding needs that are out there, that this would just be one” (Participant 19, Medical Physician, Canada)*
Provide Leadership and Advocacy	Lack of political interest in lymphoedema care among other issues		*“They've (Governmental Agencies) got to recognize it (lymphoedema), and if they recognize it, then they’ve got to come up with some means of funding to make sure that these policies get in place and that they are helping people to implement them, because it's all well and good to write a policy but, if you don't develop the strategies to help people to implement it, it's not going to happen” (Participant 19, Medical Physician, Canada)*
Support legislative frameworks	Lack of regulatory frameworks for lymphoedema care resourcing		*“Once you get people (Governmental Agencies) to recognize that (lymphoedema care), then you probably will develop the strategies and the regulatory bodies that may be necessary to provide resources or arrange for resources or an approach” (Participant 19, Medical Physician, Canada)*
**2. Health care organisation level**			
Organise and equip health care teams	Building healthcare professionals lymphoedema capabilities	Grassroots lymphoedema capacity building	*“We wanted to develop a train-the-trainer model, so we could empower the people there to basically take care of their own, and provide them the necessary resources and the education. And then phase ourselves out, essentially, as they became independent with the clinic side of things. And it was a very challenging, but very wonderfully rewarding program to be involved in” (Participant 2, Physiotherapist, USA)*
		Multidisciplinary team approach to lymphoedema care	*“… it is the physios' job, physio or occupational therapists, those who are trained in lymphoedema management. And we have very cordial relationship with onco-surgeons and the onco-nurses, as well as a physio and occupational therapists. So we work as a team and we have this Disease Management Group… So we do attend their regular meetings, so we are members of breast DMG. So we are having very cordial relationship. (Participant 10, Physiotherapist, India)*
			*“It's not rocket science. It's the basic. It depends where you go. Physiotherapists or OTs. I think if there's other musculoskeletal impairments, you need the physio because the OTs do not have that level of expertise. Nurses certainly don't have it. They're good at flagging, identifying these issues. …. I think we should just all work together” (Participant 9, Occupational Therapist, Australia)*
		Increasing the healthcare teams’ lymphedema knowledge and capabilities	*“Limited resources and limited professional skills. Definitely. But they are all keen to learn. So if you can get funding to get a program up and going they will be very attentive, but the most important thing is don't try to take their modern products over there. Use their products, learn how to use what they have there. That's a much better way to teach them” (Participant 17, Wound Nurse Consultant, Australia)*
		Developing Lymphoedema guidelines applicable to LMIC	*“I'm sure they're applicable. I think that may maybe for… I understand, well what we think the guidelines should be, should be evidence-based. And we can apply those ones, but we would be more comfortable if we are able to, let's say, test those ones and know that they actually work in our sets and then we can confidently say that these are our guidelines. But aside that, we are open to using resources from elsewhere” (Participant 1, Physiotherapist, Ghana)*
Promote continuity and coordination	Strengthening continuity and coordination for lymphoedema care	Too little sharing of clinical information to inform care	*“The other health professionals involved are only on referral like nutritionist, psychologist, because there is no formal referral from the doctor, no access to make a history so we rely on what patient is open to disclose, and ask for the name of the doctors who are involved in the care, but it's up to the patients. Very late stage though” (Participant 3, Physiotherapist, Peru)*
		Need for regular follow-up	*“And, we usually ask them to come for check-up every three months just to check. Even if the patient is discharged, I ask her to come back just for a check-up for the measurement and the texture of the arm or the leg, every three months. We do a reminder” (Participant 11, Specialist Lymphoedema Therapist, Lebanon)*
Support self-management and prevention	Effective patients’ self-management and treatment adherence	Encouraging health seeking behaviours	*“Yeah. So we try to shadow the appointments such that, first and foremost, they're not too tired and it's not too much of a burden. And sometimes when they come, they are probably unable to do two treatments and come to physio and then go to another department or other reviews at the same time. So we try to make the appointments as flexible as possible for them. So this will encourage to come regularly” (Participant 1, Physiotherapist, Ghana)*
			*“Let's say, the patient, she doesn't want to bandage on daily basis and I know that she's in the maintenance phase, if she is in the intensive phase, no, there is no negotiation” (Participant 11, Lymphoedema Specialist, Lebanon)*
		Shared decision-making and understanding patient’s preference	*“the patient, they know nothing about the lymphatic system and the lymphedema. So I start from the scratch. I have simple diagrams, sometimes I draw for the patient to explain what is the lymphatic system, its relation to the cardiac circulation and all this stuff. Then I start to explain to him what the injury caused to his lymphatic system. And I start to explain how he can make it worse and how he can make it better. Then we start the dialogue about this is a chronic condition and most of the patients are expecting that you give them a pill or you just put a machine on the leg and the problem is solved. And this really takes me a very long time” (Participant 6, Physiotherapist, Egypt)*
		Involving patient family and friends in lymphoedema care	*“And the models that I've seen that are so powerful are about community engagement, community participation, family participation. It's what I believe in so much”. “But, of course, not everybody has family. It's easy to assume that, but of course, a lot of people don't”*
			*“The model in India, for instance, is about you don't just take the patient, you take them and their family. So they come, and their family are taught how to help the patient. It's a whole family. I wish we could have some of the things that they have in our country, because we are very much about the nuclear family, and families don't … I mean, some families do, but the idea of the culture of the families, and very, very powerful one are absolutely, that's what's transformational for people (Participant 15, Nurse Consultant, UK)*
		Implementing person-centred lymphoedema care	*“…if I have a primary lymphedema patient, I have to follow the same rules. Now, depending on every patient, I might have to adjust each component of the whole CDT. A patient might be needing more compression, another patient might be needing…, but all in all, the main components are present, whether the patient is coming for secondary lymphedema or whether the patient is coming for primary lymphedema” (Participant 11, Lymphoedema Specialist, Lebanon)*
		Modifying and improvising lymphoedema care	*“But because of the materials are very costly, we use alternative options like cotton fabric and different sort of materials of similar to the proper bandages for patients to use” (Participant 3, Physiotherapist, Peru)*
			*“Very good. Most families really want to get involved and want to do as much as they can. And they actually enjoy learning how to do it. It's just getting them the resources. So for example, you can use cotton sheets, you can cut strips of a cotton sheet up and teach them how to wrap. So there are ways, they do have things, and we can just teach them how to use what they have” (Participant 17, Wound Nurse Consultant, Australia)*
		Supporting and promoting lymphoedema self-management	*“Where he has to take care of himself like skincare, like self MLD and he has to put on his stocking on a regular basis. He does this for a lifetime, it's a chronic problem. I usually see him once per month, three or four times per year” (Participant 6, Physiotherapist, Egypt)*
			*“Whether it was skin care or wound care, providing compression, modified compression … And the key, too, was not just the hygiene but the manual lymph drainage. We were able to train everybody, basically, on the essential skills to be able to manage this. And ultimately, train the patients for life, as this is something they had to manage for life” (Participant 2, Physiotherapist, USA)*
Encourage quality care through leadership and incentives	Investing in lymphoedema care	Creating an environment for patients to receive quality lymphoedema care	*So then it's about looking at what is a system of care which is really realistic and usable? Then it's looking at advocacy, empowering patients in organizations, and all … There's so many levels that we need to look at to try and make a difference” (Participant 15, Nurse Consultant, UK)*
		Need for universal health care to cover the costs of lymphoedema care, inclusive of materials	*“With the public sector, with the insurance that is fully covered by the hospital the government, it only is a service but not the materials. So the patient still needs to buy all the materials and the garments. So it's basically just paid for the service given by the physiotherapist” (Participant 4, Physiotherapist, Peru)*
**3. Community level**			
Raise awareness and reduce stigma	The burden of stigma impacts adversely on every aspect of the persons’ life	Reducing feelings of shame and embarrassment	*“…but we do experience that patients feel very much ashamed when there is something like lymphedema and due to that they are embarrassed by their body, there is a decrease in social interaction” (Participant 7, Physiotherapist, India)*
		Reducing nihilism	*The reality is, this is a condition that takes an awful lot of effort to get a change. It's of very little interest to most people, and there is a belief in the professions where you can't do anything. So then there's this apathy around it” (Participant 15, Nurse Consultant, UK)*
			*The power behind a condition is related to how the medical profession view it. Lymphedema is not the sexy, or you know? Nobody wants to work in lymphedema, it's not neuroscience or something (Participant 6, Physiotherapist, Egypt)*
		Fear and stigma	*So this huge limb which is what everybody recognizes as elephantiasis… There's a huge fear around that, there's lots of taboos around it, there's lots of beliefs. so they wait, and the patient might know early on they're getting changes, but they very often then end up with people like Doctor [XXX] out of complete desperation. (Participant 15, Nurse Consultant, UK)*
	Raising awareness of lymphoedema	Promoting understanding of lymphoedema through information	*“We have a major problem, not only in lymphedema, but in lymphedema it's much more showing, when it comes to patient education and patients having access to information.* But, to have access to information on lymphoedema, it is very difficult. *There is no direct, official, one resource of information for the patients” (Participant 11, Lymphoedema Specialist, Lebanon).*
		Encouraging and supporting health seeking behaviours	*“And we also insist one of the relatives to attend this program. So they know basically what is the signs and symptoms of lymphedema. So the moment they get any of their signs and symptoms, they don't need to go to the surgeon or oncologist first, they can directly come over to us so that we can treat them in that hospital itself” (Participant 10, Physiotherapist, India)*
			*“And then I've done other work in lymphedema, centering around and publishing … talking about the understanding of the lymphedema continuum, as we're starting to recognize that all oedema essentially is lymphedema, and what that means. So, we're trying to raise awareness about that through the work of Dr. Stanley Rockson out of Stanford University” (Participant 2, Physiotherapist, USA)*
Mobilise and coordinate resources	Promoting Peer support	Practical peer support groups	*“They have a really neat group down there. It's a women's group and they do a lot of sewing. And really, for whatever the needs are. And the idea was to see if they could come up with a way to make custom … like, sew garments for people that needed them” (Participant 2, Physiotherapist, USA)*
			*“There are various forums where the patients are connected to each other. So there is something like patient support groups of cancer support groups, from which the referrals flowing. And of course through word of mouth from patient to patient” (Participant 18, Physiotherapist, India)*
		Accessing traditional healing	*“The traditional route in many of these countries is to go to the traditional healers first” (Participant 15, Nurse Consultant, UK)*
			*“So in meeting with a doctor, they're already late in reporting, and they may have tried all forms of herbal or traditional medicine to help, and so most of the time they come in late” (Participant 1, Physiotherapist, Ghana)*
Promote consistent financing	Lymphoedema care is costly	Lymphoedema impacts on employability	*“And I also find that most of our lymphedema cases, by the time they come to us, they may have resigned, and then also many of them are people who are self-employed. So they really do not get the support of their employers. And they don't really have a company or they don't have employers taking care of them. And so they need to bear the cost themselves, many of them” (Participant 1, Physiotherapist, Ghana)*
		Lymphoedema care not covered by Universal Health Care	*“They don't actually pay anything. It's funny when the government introduced some insurance scheme, but that insurance scheme is only for public workers, people are working for the government. Essentially free of charge management, they don't even pay for it. It's free” (Participant 5, Oncology Nurse, Zambia)*

*CDT* Complex Decongestive Therapy, *ICCCF* World Health Organization’s Innovative Care for Chronic Conditions (ICCC) Framework, *MLD* Manual Lymphatic Drainage, *UK* United Kingdom, *USA* United States of America

### Joining forces to overcome lymphoedema related stigma

Stigma was seen as a key factor influencing the experiences of people living with lymphoedema. Participants reported their patients felt shame and embarrassment because of their condition and decreased social interactions due to stigmatizing from community members.

*“...patients feel very much ashamed when there is something like lymphoedema and due to that they are embarrassed by their body, there is a decrease in social interaction” (Participant 7, Physiotherapist, India).*

Participants perceived that a lack of community awareness about lymphoedema led to their patients becoming a subject of curiosity which, in turn, contributed to social avoidance.

*“… it's a big issue, in India if they (community members) see someone with a huge limb or in fact, a little bit extra swelling or with compression sleeve on…. They would come and ask them what happened to you? And then they will give their self-advice, what to do next. And this puts the patients, those who are suffering with lymphoedema into a lot of discomfort and stigma. So usually that's a big issue because always they try to hide their swelling and they don't wear a compression sleeve often if they go out, just to avoid these frequent questioning by the general public.” (Participant 10, Physiotherapist, India).*

Participants also reported encountering cultural beliefs that the condition was caused by sorcery or repayment of a past sin, or that lymphoedema was contagious, further adding to stigma.

*“I think that's probably a lot of things. Some of it could be knowledge, in some locations there is a concern that lymphoedema is caused by sorcery” (Participant 13, HIC Epidemiologist, reporting on lymphoedema care in Benin, Mali, Tanzania and India).*

*“Their culture… there is a lot of voodoo and different beliefs there that maybe they were possessed, or that they had done something wrong. People didn't understand that it wasn't contagious ... all these different things. So we had to demystify it a little bit. (Participant 2, HIC physiotherapist, reporting on lymphoedema care in Haiti and India).*

In some countries, gender roles were said to be important in determining who sought and received lymphoedema care. Men were generally considered more likely to go for treatment than women because their partners tended to be more supportive.

*“… treatment seeking based on gender bias. Because if a male having a lymphoedema, then the family would aggressively come forward and seek for treatment. And the wife would be always there for helping them, but not the same for a female suffering from lymphoedema. It's the case, their voice [the voice of the men], they come forward for treatment late and families may not be willing to spend much money on them. And yeah, that's a big issue” (Participant 10, Physiotherapist, India).*

### Building workforce lymphoedema capabilities

#### Develop health professional knowledge

The lack of community awareness and support for lymphoedema was reported to be compounded by a belief prevalent among health professionals that the condition could not be successfully managed.

*The reality is, this is a condition that takes an awful lot of effort to get a change. It's of very little interest to most people, and there is a belief in the professions where you can't do anything. So then there's this apathy around it” (Participant 15, HIC Nurse Consultant, reporting on lymphoedema care in South Africa, India, Uganda, Tanzania, and Sierra Leone).*

Participants emphasised that a lack of skilled health care professionals was a key barrier to delivering optimal care in LMIC and indicated the need to build capacity by means of a train-the-trainer model to reduce reliance on international expertise.

*“…training more [local] people and they can go out and create master trainers who then will train health care providers who will then train people who are the clients, the recipient” (Participant 13, HIC Epidemiologist, reporting on lymphoedema care in Benin, Mali, Tanzania, and India).*

*“We wanted to develop a train-the-trainer model, so we could empower the people there to basically take care of their own, and provide them the necessary resources and the education. And then phase ourselves out, essentially, as they became independent with the clinic side of things. And it was a very challenging, but very wonderfully rewarding program to be involved in” (Participant 2, HIC Physiotherapist, reporting on lymphoedema care in Haiti and India).*

Where expertise was not available within the country to provide training, participants emphasized that expert teams from HIC should aim to build a sustainable workforce after their time-limited involvement was over, as well as one that was sensitive to local contexts, including culture.

*“[We need to be saying] ‘we’re not here to give you a fish, we're here to teach you how to fish’. And that's the key thing. But the point that I also want to make is that it's an ongoing process. And you really have to work so hard on developing those local champions. And the problem is you develop these, and they're really very good and they move things along. But when they're competent, they do well, they tend to move on to other things” (Participant 19, HIC Medical Physician, reporting on lymphoedema care in Haiti, Uganda, and Brazil).*

Participants stressed the need for health care professionals in LMIC to become acquainted with available resources and tools in handling the condition.

*“Limited resources and limited professional skills. Definitely. But they are all keen to learn. So if you can get funding to get a program up and going they will be very attentive, but the most important thing is don't try to take their modern products over there. Use their products, learn how to use what they have there. That's a much better way to teach them” (Participant 17, HIC Wound Nurse Consultant, reporting on lymphoedema in Fiji, Cook Island, Solomon Island, Papua New Guinea, Bangladesh, and Indonesia).*

Participants confirmed that health professionals required continuing education and support. Participants indicated that treatment was not possible under rural conditions due to limited resources in Pacific and Africa Regions, requiring capacity building.

*“So it's, so the gap is: one, is the knowledge of the health care practitioners; two, is the availability of the materials to treat and manage these conditions; and three, the treatment management itself is not very friendly in a very rural conditions of these countries” (Participant 14, HIC Dermatologist, reporting on lymphoedema care in West Africa).*

#### A commitment to multidisciplinary team care

The importance of a coordinated multidisciplinary team approach to lymphoedema care was emphasized in order to address the condition’s multidimensional impacts.

*“…sometimes depending on the condition, we let the dietician come in to help with some diet modification. And sometimes we have to bring in the psychologist to help them accept all that they have to do deal and then make our treatment also more friendly and more meaningful for them (Participant 1, Physiotherapist, Ghana).*

Unfortunately, however, differing professional perspectives and a lack of team mentality were said to create challenges to a multidisciplinary approach:

*“*So *what happened in the real life is that the surgeon finished his surgery and the oncologist finished with the radiotherapy and his chemotherapy [for people with cancer-related lymphoedema]. It's like I don't want to see the patient again. I have nothing to do with the lymphoedema. Nobody [health worker] has to ask me about the lymphoedema. Nobody talks about the scar or the lymphoedema. This is not my concern, this is not my part and I'm not interested to help the patient” (Participant 6, Physiotherapist, Egypt).*

Participants also identified important roles for other support services, including hospital navigating services, community health workers, and voluntary organizations who helped in lymphoedema management in Africa, and South America.

*“And actually, and of course, the traditional, I mean the community health workers, volunteers, support workers, they also sometimes are involved, but really actually they are very good. Sometimes very good in dressing change and then managing these patients” (Participant 14, HIC Dermatologist, reporting on lymphoedema care in Ivory Coast, and Ghana).*

#### The need for lymphoedema guidelines applicable to LMIC

Participants perceived there to be a total absence of guidelines for lymphoedema care in LMIC.

*“So, for the guidelines, there are no (LMIC) guidelines” (Participant 11, Specialist Lymphoedema Therapist, Lebanon).*

While some participants reported not using any guidelines at all, several indicated that they used guidelines developed for HIC but expressed a need for these to be adapted to low resource settings.

*“… we can apply those ones (HIC guidelines), but we would be more comfortable if we are able to, let's say, test those ones and know that they actually work in our setting, and then we can confidently say that these are our guidelines. But, aside that, we are open to using resources from elsewhere” (Participant 1, Physiotherapist, Ghana).*

Most participants seemed unaware of the WHO guideline on wound and lymphoedema management [21], and others indicated was narrow in content.

*“We haven't seen any guidelines (WHO). We only apply knowledge from the school. But there are no guidelines per se, no some extra piece that someone can follow. No, we don't have that (WHO guideline), but we just use the knowledge that we've acquired from our schools” (Participant 5, Oncology Nurse, Zambia).*

### Partnering with patients and families to support self-management

Participants reported that effective lymphoedema care was dependent on patients having positive and established relationships with health professionals to set shared goals and enable access to resources to enable self-management. Appropriate treatment planning that included setting clear goals and ensuring follow ups were suggested as being critical to improving care and outcomes in LMIC.

*“And patients, they can be taught a lot of ways to manage this themselves. And that's ultimately the goal. We help reduce it, and then they maintain it. That's really the position we took when we tried to manage it.” (Participant 2, HIC Physiotherapist, reporting on lymphoedema care in Haiti, and India).*

Participants reported that they sometimes even gave free consultations to enhance self-management.

*“We used to do a lot of free consultations for them. And that was our prerogative. It was not exactly allowed. So sometimes we do, when we do the education, we usually not let them pay for it” (Participant 1, Physiotherapist, Ghana).*

#### The need for a person-centred approach

Participants stressed the need for person-centred care that focussed on each individual’s care needs. While participants felt there were standard steps to follow in lymphoedema care, they tailored treatment for each individual and prescribed regimen based on the patient’s condition and presentation stage.

*“…if I have a primary lymphoedema patient, I have to follow the same rules. Now, depending on every patient, I might have to adjust each component of the whole CDT. A patient might be needing more compression, another patient might be needing…, but all in all, the main components are present, whether the patient is coming for secondary lymphoedema or whether the patient is coming for primary lymphoedema” (Participant 11, Specialist Lymphoedema Therapist, Lebanon).*

Participants indicated that they had to work closely with patients in planning and modifying their management to maximize individual outcomes.

*“…Let's see what treatment is best suited to your needs." So, I know what is a gold standard treatment, but does that match with your life, and your expectations, and your reality? Then I would plan, I would say, "If we can work together, we can make differences in your life" (Participant 15, HIC Nurse Consultant, reporting on lymphoedema care in South Africa, India, Uganda, Tanzania, and Sierra Leone).*

#### The importance of clear communication to help patients understand their condition and self-management

Participants indicated the need to engage patients in clear communication about their condition to help them understand the management approach. Participants indicated that discussing patients’ conditions with them help to debunk any misunderstandings they might have.

*“The patient, they know nothing about the lymphatic system and the lymphoedema. So I start from the scratch. I have simple diagrams, sometimes I draw for the patient to explain what is the lymphatic system, its relation to the cardiac circulation and all this stuff. Then I start to explain to him what the injury caused to his lymphatic system and how he can make it worse or better. Then we start the dialogue about this chronic condition, which takes a lot of time. Most of the patients are expecting a pill or you just put a machine on the leg and the problem is solved” (Participant 6, Physiotherapist, Egypt).*

Participants sometimes provided information leaflets to help patients understand their condition better.

*“…we have a patient information leaflet that we give to them to take along and also we actually, before we start their managed treatment” (Participant 1, Physiotherapist, Ghana).*

Participants also used social media to educate patients on lymphoedema.

*“…Through doctors and me, and I have some brochures, I have my Instagram account, I have my social media things that I can give all the information for my patients” (Participant 8, Physiotherapist, Bahrain).*

#### Promoting adherence to management

To encourage patients’ attendance to treatment, participants mentioned several strategies including planning schedules with patients, making flexible appointments and making it easy for patients to come for treatment and to allow some adjustments to promote better adherence to an agreed management plan.

*“So we try to shadow the appointments such that, first and foremost, they're not too tired and it's not too much of a burden. And sometimes, they are probably unable to do two treatments and come to physio and then go to another department or other reviews at the same time. So we try to make the appointments as flexible as possible for them. So this will encourage to come regularly” (Participant 1, Physiotherapist, Ghana).*

If patients’ needs were sufficiently met, they were more likely to be motivated to self-manage their condition.

*“The last thing patients want to do is to put on bandages. And that was probably a logistical challenge, as well. But when they [health workers] explained to them, the patients saw finally, that they could get a reduction in their lymphoedema, and it could be maintained better by wearing compression, they typically opted to wear the compression. That was a reward in and of itself” (Participant 2, HIC Physiotherapist, reporting on lymphoedema care in Haiti, and India).*

However, poor support systems and limited time for consultations and follow-ups was said to contribute to many LMIC patients having to cease their lymphoedema care or resort to alternative, less effective ways of managing their condition.

*“I think sometimes the treatment can be discontinued because they go back to their villages. And there's only one nurse in the village for many, many people. So some places there was one nurse for 500 people. So then the time of the nurse, and they're not all, they don't all work the same hours that we would work, for example. So ‘if you don't get your treatment by three o'clock, well, you're not getting treated today cause I'm finished’ - that sort of attitude” (Participant 17, HIC Wound Nurse Consultant, reporting on lymphoedema care in Fiji, Cook Island, Solomon Island, Papua New Guinea, Bangladesh, Indonesia).*

Participants suggested that patients became especially motivated by seeing improvements in their condition.

*“But when the patients saw finally, that they could get a reduction in their lymphoedema, and it could be maintained better by wearing compression, they typically opted to wear the compression. That was a reward in and of itself” (Participant 2, HIC Physiotherapist, reporting on lymphoedema care in Haiti, and India).*

Participants highlighted that multiple competing interests for patients could distract from their self-management, and put in place various strategies to overcome this barrier, such as sending reminder messages as well as having regular consults planned for patients.

*“And, we usually ask them to come for check-up every three months just to check. Even if the patient is discharged, I ask her to come back just for a check-up for the measurement and the texture of the arm or the leg, every three months. We do a reminder” (Participant 11, Specialist Lymphoedema Therapist, Lebanon).*

#### Adapting management to the resources available to patients

Participants unanimously agreed that the standard model of lymphoedema care should include available resources for lymphoedema management. However, due to limited resources, they had to modify the management for patients who could not afford the necessary materials.

*“Most families really want to get involved and want to do as much as they can. And they actually enjoy learning how to do it. It's just getting them the resources. So for example, you can use cotton sheets, you can cut strips of a cotton sheet up and teach them how to wrap. So there are ways, they do have things, and we can just teach them how to use what they have” (Participant 17, HIC Wound Nurse Consultant, reporting on lymphoedema care in Fiji, Cook Island, Solomon Island, Papua New Guinea, Bangladesh, Indonesia).*

Participants indicated shortage in supply in most materials. Participants had to improvise and, in some cases, resorted to repurposing materials.

*“We either had to find local resources that were sustainable and reliable ... or what we did is, a lot of times, we would just ... And we would repurpose compression, that we would teach them how to wash and take care of the bandages so that they could be reused” (Participant 2, HIC Physiotherapist, reporting on lymphoedema care in Haiti, and India).*

#### Involving patient family and friends in lymphoedema care

Participants indicated that support from families and friends was especially important to the care of their person with lymphoedema.

*“And the models that I've seen that are so powerful are about community engagement, community participation, family participation. It's what I believe in so much”. “But, of course, not everybody has family. It's easy to assume that, but of course, a lot of people don't” (Participant 15, HIC Nurse Consultant, South Africa, India, Uganda, Tanzania, Sierra Leone).*

Participants indicated that the friends and families not only provided emotional support and served as motivation for patients to attend appointments but could also provide financial support and could be involved in hands-on care if trained.

*“Very good. Most families really want to get involved and want to do as much as they can. And they actually enjoy learning how to do it” (Participant 17, HIC Wound Nurse Consultant, reporting on lymphoedema care in Fiji, Cook Island, Solomon Island, Papua New Guinea, Bangladesh, Indonesia).*

## Discussion

This study is the first to explore barriers and facilitators to lymphoedema care in LMIC. Application of the ICCC Framework provides insights into ways to improve lymphoedema care in low resource settings, as well as opportunities for further research.

Raising awareness and reducing stigma appears to be especially important, given stigma often arises from a combination of cultural factors and limited understanding of the condition that prevent help-seeking and contribute to social isolation and functional impairment. Previous research confirms that people with lymphoedema in some LMIC may be perceived to have suffered a condition caused by “evil spirits” [22]. Demystifying the stigma associated with lymphoedema in LMIC must be a consensus effort. In a review of literature focusing on stigma reduction strategies for mental health conditions in LMIC, Javed et al. [23] identified a need to implement health literacy programmes, engage the media on relevant topics, and combat the use of discriminatory and stigmatizing terms. Future research is needed to evaluate the generalizability of such strategies for lymphoedema-related stigma.

While lymphoedema-related knowledge may be limited both among people living with the condition and health care professionals working outside of cancer, there is evidence that building workforce capacity has a positive impact. A case study in Ghana and Malawi has shown that, when given the appropriate tools and education, community health workers become well equipped in early identification of lymphoedema, reducing morbidity and improving quality of life [24]. Our study suggests that community health workers may be even more effective when working in collaboration with hospitals and voluntary organizations. Without such interventions, our results support others in highlighting that lymphoedema is often diagnosed at a late stage in LMIC due to the limited resources, especially when from causes other than cancer, including lymphatic filariasis, podoconiosis and trauma [25].

Critical components from the ICCC Framework that were less well represented in our data tended to relate to “positive policy environment”. Where policy environment was referred to, there was an emphasis on the need for governments to first and foremost recognize lymphoedema care as a priority, as well as consideration of the limited capacity that high-level policies might have to improve care on the ground without the required resources and implementation strategies. These findings accord with previous commentary on the need to decentralize health system improvements in LMIC to non-managerial groups such as health care professionals and communities [26]. If health care systems in LMIC are to improve, then coherent action across all sectors of national, regional, and community levels, including finance, housing, education and training, transport and health, is needed to achieve equity and access [27].

A key barrier highlighted by participants in the current study is the lack of guidance available to health care professionals in LMIC to identify, assess and manage lymphoedema. There appears to be only one relevant guideline published by the WHO, which focuses largely on management of wound and lymphoedema caused by filariasis [21]. Most participants seemed unaware of this guideline, and others indicated was narrow in content. Participants generally reported depending on HIC guidelines that were not always applicable to the limited resources they had available. For example, participants reported that many patients will be unable financially to meet the high cost of bandages and compression garments, and also lack transportation to consults – a problem also commented on by previous authors [28]. Our study highlights the importance of health care professionals partnering with patients to support their self-management and adapt plans to be within their means. Our findings are consistent with studies from HIC which suggest that self-management is facilitated by engaging family support systems, clear communication, and employing a person-centred care approach to lymphoedema management [14].

Notable gaps that should be addressed by future research include understanding patient perspectives on lymphoedema-related care delivery and self-management in LMIC. Taken together with the perspectives of professionals canvassed in the current study, this might further inform needs assessment of persons living with lymphoedema as well as interventions for this patient group. Secondly, in the absence of guidelines specific to LMIC, there is the need to inform adaptations of HIC resources for both clinicians and patients.

Findings from the current study are limited by the fact we could not represent experiences from all or even a majority of LMIC and, even within each LMIC, experience was focused on one region or city. Experience of lymphoedema was also more weighted towards cancer than other causes relative to their prevalence in LMIC. However, we were able to include a diversity of experience in lymphoedema care across global regions and health care disciplines and found remarkable consistency in the barriers/facilitators reported, increasing confidence in the generalizability of our findings.

It should be noted that almost half of our sample were from HIC so were unlikely to have the same insight into local issues and the way to solve these as participants who were from the LMIC they were interviewed about. Increasingly, there is emphasis on development initiatives in LMIC of all kinds being driven by local people because they should set the priorities and can build capacity for sustainable change as well as ‘ownership’ and acceptance of initiatives by local communities [29, 30]. At the same time, the HIC participants contributed a high level of expertise on lymphoedema and often had experience across more than one LMIC to draw on regarding which solutions might be transferable versus context dependent.

Majority of participants in this study are physiotherapists despite the broader recruitment strategy employed. This is consistent with a paucity of specialised medical personnel reported by Schulze et al. [31] in their worldwide assessment of lymphoedema care. At the same time, participants often provided insight into disciplines and services for lymphoedema care beyond the one they were directly involved with.

As a trained physiotherapist from Ghana, ET’s ‘insider’ position was a strength in that he could compare participant reported barriers with his own experience to assess whether they might be generalizable or specific to the LMIC the participant reported on [32]. On the other hand, there was a risk that ET’s experience might lead his interpretation to a greater extent than participants’ intended meaning [33]. This was safeguarded by the broader research team’s involvement in analysis, who critiqued ET’s interpretations and helped to organise the data into the main concepts.

## Conclusion

A number of barriers to lymphoedema care in LMIC have been identified to include limited resources, absence of specialists, and lack of regulatory frameworks, increased cost, and increased stigma. Improving lymphoedema care in LMIC involves joining forces to overcome lymphoedema related stigma, building workforce lymphoedema capabilities, and partnering with patients and families to support self-management. Improving lymphoedema care in LMIC requires a concerted efforts from all stakeholders including healthcare team, patients and their families. Future research should aim to understand patient perspectives on lymphoedema care and inform adaptation of existing HIC lymphoedema resources for LMIC contexts.

## Supplementary Information

**Additional file 1.**

## Data Availability

The de-identified datasets analyzed in the current study are available from the corresponding author on reasonable request.

## Abbreviations

HIC: High Income Countries
ICCC: Innovative Care for Chronic Conditions
LMIC: Low- and Middle – Income Countries
WHO: World Health Organization
