# Supplementary material for: Improving care for cancer-related and other forms of lymphoedema in low- and middle-income countries: a qualitative study
Source: BMC Health Serv Res. 2022 Apr 8;22:461. doi: 10.1186/s12913-022-07840-7 (PMC8990607; doi:10.1186/s12913-022-07840-7)
Supplement: Supplementary file 1 — Additional file 1. [file 12913_2022_7840_MOESM1_ESM.docx]

**Supplementary Table 1: List of Organisations**

| **S/N** | **Organisation** | **Location** |
| --- | --- | --- |
| 1. | Asia Pacific Journal of Cancer Prevention | Iran |
| 2. | Asia-Pacific Journal of Oncology Nursing | USA |
| 3. | International Society of Nurses in Cancer Care (ISNCC) | Canada |
| 4. | Lymphology Society of India | India |
| 6. | European Wound Management Association | Denmark |
| 7. | Wounds International | UK |
| 8. | Lymphoedema Network Northern Ireland | Northern Ireland |
| 9. | Indian Association of Palliative Care | Indian |
| 12. | Brazilian Society of Surgical Oncology (SBCO) | Brazil |
| 13. | Philippine Cancer Society | Philippine |
| 14. | Cancer Society Nepal | Nepal |
| 15. | Indian Cancer Society | India |
| 16. | Egyptian Society of Surgical Oncology | Egypt |
| 17. | Myanmar Oncology Society | Myanmar |
| 18. | Cancer Foundation of China | China |
| 19. | Sri Lanka Cancer Society | Sri Lanka |
| 20. | National Cancer Society of Malaysia | Malaysia |
| 21. | National Cancer Institute - Thailand | Thailand |
| 22. | American Cancer Society Mexico | Mexico |
| 23. | Fiji Cancer Society | Fiji |
| 24. | Cancer Society in Mauritius | Mauritius |
| 25. | Swaziland Breast Cancer Network (SBCN) | Swaziland |
| 26. | National Cancer Council of Mongolia | Mongolia |
| 27. | Cancer Institute, Imam Khomeini Medical Center | Iran |
| 28. | Guyana Cancer Prevention Society | Guyana |
| 29. | Cancer Association of South Africa | South Africa |
| 30. | Turkish Society of Medical Oncology | Turkey |
| 31. | Lymphatic Education & Research Network | USA |
| 32. | Australasian Lymphology Association (ALA) | Australia |
| 33. | African Palliative Care Association | Uganda |
| 34. | International Lympheedema Framework (ILF) | UK |
| 35. | National Lymphedema Network | USA |
| 36. | Cancer Center, Physical Medicine and Rehabilitation Department, Mayo Clinic | USA |
| 37. | World Alliance for Wound and Lymphedema Care (WAWLC) | Switzerland |
| 38. | World Health Organization (WHO) | Switzerland |
| 39. | World Confederation for Physical Therapy (WCPT) | UK, International |
